# Reducing the Impact of Tinnitus on Children and Adolescents' Lives: A Mixed-Methods Concept Mapping Study

**DOI:** 10.1155/2021/5534192

**Published:** 2021-05-31

**Authors:** Susan Tegg-Quinn, Robert H. Eikelboom, Christopher G. Brennan-Jones, Syndon Barabash, Wilhelmina H. A. M. Mulders, Rebecca J. Bennett

**Affiliations:** ^1^Ear Science Institute Australia, Subiaco, Australia; ^2^School of Human Sciences, The University of Western Australia, Nedlands, Australia; ^3^Ear Sciences Centre, The University of Western Australia, Nedlands, Australia; ^4^Department of Speech Language Pathology and Audiology, University of Pretoria, Pretoria, South Africa; ^5^Ear Health Group, Telethon Kids Institute, The University of Western Australia, Nedlands, Australia; ^6^Division of Paediatrics, UWA School of Medicine, The University of Western Australia, Nedlands, Australia; ^7^Department of Audiology, Perth Children's Hospital, Perth, Australia; ^8^Syndon Barabash, Iris Family Medicine, Glen Iris, Australia

## Abstract

**Objectives:**

To generate a conceptual framework describing what is done to reduce the impact of chronic tinnitus on the lives of children and adolescents.

**Design:**

Views and experiences of 32 adults from two participant groups informed this concept mapping study: (i) a tinnitus group (adults who experienced tinnitus during childhood/adolescence, and primary carers of children/adolescents with tinnitus) and (ii) a clinicians' group (clinicians who provided care for children/adolescents with tinnitus). Participants produced statements describing what is done to reduce the impact of chronic tinnitus on the lives of children and adolescents who experience it. Through grouping and rating processes, they identified key concepts and inferred their associated benefit.

**Results:**

The participants generated 102 unique statements across four concepts: (1) *Education, Support, and Counselling*; (2) *Support from Parents and Teachers*; (3) *Clinical Assessments and Management*; and (4) *Self-Management Techniques*. Many statements highlighted the need for child-friendly and patient-centred care. Adults with personal experience of childhood tinnitus tended to perceive many of the statements as more beneficial than did the clinician group.

**Conclusions:**

Although many children will develop management strategies to assist them with their tinnitus, both the adults who experienced tinnitus as children and their parents valued strategies involving clinical care, knowledge, and expertise. Participants from the tinnitus group perceived a greater degree of benefit associated with strategies from all four clusters than the clinicians' group. However, both groups perceived the greatest degree of benefit as being associated with activities and strategies within the *Education, Support, and Counselling* and the *Clinical Assessments and Management* clusters. Both groups identified that recognising the occurrence of tinnitus for children and adolescents, acknowledging the potential for associated distress, and initiating clinical care provide the nexus of effective management. Addressing the concerns and needs of parents was also perceived as valuable; hence, approaching the management of tinnitus during childhood and adolescence from a family-centred care framework is recommended.

## 1. Introduction

Tinnitus during childhood and adolescence (hereafter referred to as tinnitus during childhood) is defined as the perception of sound without an external stimulus [1–3]. Although some studies have suggested that tinnitus occurs at least as frequently in children as it does in adults [4, 5], significant variations exist between studies estimating both prevalence and associated distress. Prevalence rates of between 4.7% [4] and 54.7% [5] have been reported for tinnitus in children with normal hearing and up to 66% [6] for children with varying degrees of hearing loss. Baguley et al. [1] described children as rarely suffering from tinnitus while several other studies have described as many as 40% of children experiencing distress in association with their tinnitus [2, 7]. These differences may arise from (1) methodological differences between studies in regard to the way they elicit and measure information about tinnitus, (2) low numbers of children presenting in clinical facilities seeking assistance for their tinnitus, (3) low awareness levels of paediatric tinnitus amongst general practitioners, and (4) difficulties experienced by children in communicating their experience of tinnitus [1, 8, 9].

Tinnitus with varying degrees of associated distress has been identified as occurring during childhood, and multidisciplinary attempts to manage and ameliorate it have been identified as occurring within clinical settings in an *ad hoc* manner [10]. Smith et al. [10] in their scoping review of clinical services for children with tinnitus identified these to include medical interventions for underlying ear, nose, and throat conditions, hearing aids to address associated hearing loss, masking devices for sound enrichment, hearing protection, counselling in the form of reassurance and/or tinnitus education, narrative therapy, and psychological assistance. In a research environment, Tinnitus Retraining Therapy (TRT) with sound enrichment through hearing aids and/or noise generators [7, 11, 12], counselling [12, 13], and amplification for the suppression of tinnitus in children with hearing loss [3] have been reported.

Understanding how strategies have been developed for or have been adapted to children is imperative for understanding their suitability for this population. For example, Bartnik et al. [7] and Bae et al. [11] both undertook retrospective studies exploring the application of TRT [14] in children, yet neither adequately explained whether nor how TRT was modified or adapted prior to application in a paediatric cohort. Furthermore, neither described nor explained how the hearing aids or noise generators were to be used for sound enrichment. Similarly, both Bae et al. [11] and Lee et al. [13] undertook studies exploring the application of tinnitus counselling to ameliorate tinnitus distress in children. Bae et al. [11] described having provided a single counselling session providing reassurance, an explanation of how tinnitus relates to the auditory system, and instructions for avoiding precipitating factors to 62 children with mild tinnitus and ascribed benefit to their intervention. Lee et al. [13] reviewed the benefit of tinnitus counselling and education on tinnitus for 18 children aged 4–19 and their parents but did not describe what was involved in the tinnitus counselling and education. These study designs raise concerns as to how closely the interventions delivered adhere to previously described protocols for TRT or the content recommended for tinnitus counselling. They also raise concerns as to the suitability of the interventions to the children's needs. Interventions need to be tailored to meet children's education, developmental, and language levels [15–18]. While TRT and tinnitus counselling have been developed for adults and have theoretical underpinnings for the content and recommended durations, it is difficult to ascertain what intervention has indeed been provided to the children, whether what has been applied is developmentally suitable, and whether the content was relevant to children's needs and experiences.

Although children experience tinnitus, they may not experience it in the same manner as adults and applying methods designed for adults may fail to meet the needs of children and their families [15, 17, 19]. The impact of tinnitus during childhood can be different to that of adults, with the impact influenced by the interplay between the sound they hear, their interpretation of the sound, and the physical and emotional effects they experience in association with the tinnitus [20]. Thus, the strategies required to manage tinnitus in childhood likely need to be specifically designed for children. Currently, little is known about the strategies children implement before seeking clinical assistance and how they perceive the benefit of these strategies. Accordingly, the aim of this study was to identify the range of strategies be they self-developed, clinically recommended and delivered, or otherwise that were employed to reduce the impact of chronic tinnitus during childhood, from the reflections of those who have experienced tinnitus during childhood, their caregivers and clinicians involved in the care of children who experience tinnitus. A secondary aim was to ascertain the range of perceived benefit associated with each of the strategies identified.

## 2. Materials and Methods

This paper presents the third study that forms part of a larger three-question concept mapping project undertaken to better understand the lived experience of chronic tinnitus during childhood. The first study investigated “What does tinnitus sound and feel like to the children and adolescents who experience it?” [20]. The second study explored “What parts of children's and adolescents' lives are affected by chronic tinnitus?” [21]. This third study explores “What is done to reduce the impact of chronic tinnitus on children's and adolescents' lives?”

Concept mapping as a research technique draws upon the understandings and perspectives of participants who have experience of the topic being investigated to produce an in-depth qualitative exploration that is then combined with quantitative analysis [22–24]. The concept mapping research process was chosen because it facilitates fresh exploration of the research topic from the perspectives of those who have experience of it without undue influence from researchers, thereby encouraging fresh insights and novel perspectives which are especially valuable in areas such as tinnitus during childhood where there is a paucity of research [22, 25–29]. Three group stages of brainstorming, grouping, and rating are used to collect the participants' experiences while statement pooling and refining and quantitative data analysis were applied to analyse and interpret these insights to produce a conceptual framework [22, 25, 30]. The brainstorming stage provides a qualitative data collection method which encourages the participants to generate statements in response to the research questions thereby sharing their experiences, views, and opinions. The grouping stage involves the participants grouping all the statements generated during the brainstorming stage into groups in a way that makes sense to them, while the rating stage allows the participants to rate each statement using a scale suitable for the topic under investigation. The results of these three stages are then combined with qualitative analysis and assist the research team to develop a fuller understanding of the context around the topic being investigated as well as facilitating the identification of insights and interrelationships, thereby improving both the validity of the research findings and enhancing the applicability of any recommendations arising from the study [23, 25, 31].

Thirty-Two participants generated the brainstorming statements for all three questions in their first session. These sessions were undertaken either via teleconference where the researcher transcribed each participant's statements verbatim or participants directly entered their responses into an online Concept Systems portal (Concept Systems Incorporated 2011). Subsequently, 24 participants (22 original participants and 2 additional participants) undertook the grouping and rating activities for this question again using the online portal or through postal packs. Participants were randomly allocated to the grouping and rating activities for one of the three questions. Upon completion of their initial allocated tasks, they were invited to undertake the grouping and rating activities for either of both of the other questions. This process was undertaken to reduce participant burden. Data analysis for each of the three questions was undertaken separately and has been presented as three separate manuscripts.

The processes applied during this study followed those reported by Tegg-Quinn et al. [20] and are summarised below and in Table 1.

### 2.1. Participants

Thirty-two adult participants represent two participant groups: (i) a tinnitus group of adults who had experienced tinnitus as children (*n* = 10), as well as the primary carers of children with tinnitus (*n* = 10), and (ii) a clinicians' group of clinical professionals who care for children with tinnitus contributed to this study (*n* = 12). The 10 adults who experienced tinnitus as children (3 males and 7 females) were aged between 19 and 62 years (mean 34.9, SD 15.14). The age of tinnitus onset ranged from 4 to 18 years, and they were from Australia, Portugal, and the USA. The 10 primary caregivers (1 male and 9 females) were aged 28 to 55 years (mean 44.73, SD 7.67) and were from Australia and the United Kingdom. The 12 clinicians (1 male and 11 females) were aged between 34 and 56 years (mean 46.8, SD 7.99). They were from the United Kingdom, Denmark, and Australia. The clinicians represented three different professions: one ear, nose, and throat specialist, one psychologist, and ten audiologists. Their experience ranged from less than 5 years to over 20 years working in the field of paediatric tinnitus. Each clinician had assisted a minimum of five children with tinnitus, and the majority had assisted over 20.

The adults who had experienced tinnitus during childhood and the primary caregivers' responses were combined into one patient group as they both have lived experience of tinnitus during childhood. The primary carers were asked to provide their perspectives of their children's experiences. Adults who had experienced tinnitus as children were selected to inform this study because they have had the experience as a child with the maturity and perspective of adulthood to reflect upon their experiences. Previous studies have demonstrated that adults are able to accurately relate salient autobiographical childhood events without influence and as such were deemed ideal informants for this study [32–34]. Their perspectives and experiences were ideally combined in the concept mapping strategy to develop rich insights.

Diversity of participants' experiences, ages, and countries of origin was sought to promote the generation of varied insights and to produce a comprehensive representation and exploration of the study's question [26, 28, 29]. All participants were 19 years of age or older.

Participants were recruited using a combination of purposive and voluntary response nonprobability sampling methods via recruitment flyers posted on online international tinnitus chatrooms and forums and national tinnitus association websites in Australia and the United Kingdom. Recruitment emails were sent to universities, clinical and professional networks. All potential participants who met the eligibility criteria provided informed and written consent. No limits were placed on location or country of origin, however, of the participants who initially expressed interest only those from the listed countries participated.

### 2.2. Procedures

Human ethics approval was granted through the Human Research Ethics Office, The University of Western Australia (RA/4/201/4274). Multiple participation methods were included to encourage in-depth representation of a diverse range of views and experiences without compromising the reliability and validity of the study's findings [23, 26, 28, 29].

### 2.3. Data Analysis

Once all brainstorming, grouping, and rating data (stages 1, 2, and 3; Table 1) had been entered into the Concept Systems portal (Concept Systems Incorporated 2011), it was checked to ensure that each participant's grouping and rating data were coded according to their participation group (tinnitus or clinician). Data analysis was then undertaken using the Concept Systems software and SPSS (stage 4; Table 1).

A point map was generated using multidimensional scaling, with the proximity of points to one another representing how frequently the participants grouped the statements together. The reliability of the multidimensional scaling was established through calculating the stress index. Concept maps with a good level of reliability produce stress indices between 0.205 and 0.365, with lower scores indicating a greater degree of reliability [30].

The results of hierarchical cluster analysis are represented through the production of cluster maps. The cluster map identified key concepts unveiled during hierarchical cluster analysis. Each cluster represents a concept with the boundaries illustrating both which statements fall within each cluster and their relative location compared to others [27]. While several cluster maps may be generated for each data set, the research team determined which map best represented the participants' responses. The content of the clusters and cluster bridging scores guided the researchers' decision. Bridging scores indicated how frequently statements are grouped together, the anchor statements for each cluster and which statements bridge between the clusters. Lower bridging scores indicate that the statements are grouped together frequently. High bridging scores indicate that the statements are grouped together less frequently and consideration should be given to whether a greater number of clusters may better represent the data [27].

Establishing the final concept map's reliability and ensuring that it was representative of both participant groups was assessed using two approaches. Initially, the consistency in the grouping data from the tinnitus group was compared to the grouping data from the clinician's group by creating a concept map for each group and performing a split-half reliability measure using the concept mapping software followed by calculating and a Spearman-Brown coefficient split-half reliability test using SPSS [23]. As scores greater than 0.7 on the Spearman-Brown coefficient split-half reliability test of unequal lengths suggest that there is a strong degree of correlation between the groupings of the participant groups, the data were able to be combined to produce one representative concept map [35]. Second, a Spearman-Brown coefficient split-half reliability test of unequal lengths was applied to the participant's grouping data split into two random groups [36].

## 3. Results

### 3.1. Brainstorming and Grouping

The brainstorming phase generated 169 raw statements describing what is done to reduce the impact of chronic tinnitus upon the lives of children and adolescents. These statements were refined to 102 statements (Supplementary file 1) which were grouped and rated by 24 participants (20 original and 4 additional participants) into groups of between three and 22 statements (mean 7.58, SD 4.18).

**Figure 1:**
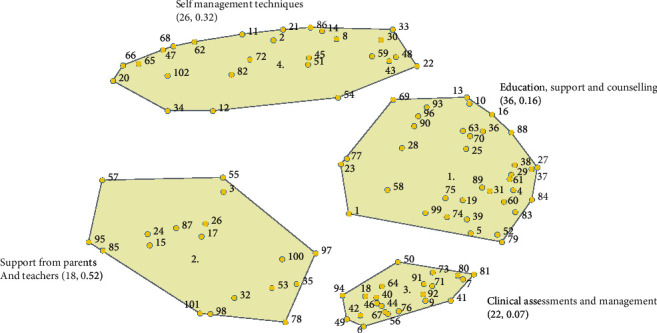
Concept map for the four clusters describing “What is done to reduce the impact of chronic tinnitus on the lives of children and adolescents?” The stress index score is 0.28, and the Spearman-Brown split-half reliability of unequal length score is 0.77. The number of statements in the cluster and the bridging scores are provided in brackets.

It was determined that a four-cluster map best represented the participants' responses (Figure 1). The four-cluster concept map had a split-half reliability score of 0.768, indicating a high degree of internal consistency [35]. The four concepts described were named: *Education, Support, and Counselling*, *Self-Management Techniques*, *Clinical Assessments and Management*, and *Support from Parents and School.*

#### 3.1.1. Education, Support, and Counselling (36 Statements, Largest Cluster)

Statements within this cluster were centred around four general themes: (1) attempts to understand children's experiences (*n* = 4); (2) assisting children to understand their tinnitus and assisting them to adopt different perspectives or ways of thinking about their tinnitus (*n* = 12); (3) ensuring that the child feels safe, supported, and reassured (*n* = 11); (4) teaching them methods to manage both their responses to their tinnitus and factors that may exacerbate their distress (*n* = 9).

#### 3.1.2. Self-Management Techniques (26 Statements)

Statements within this cluster identified methods and strategies children may use to self-manage their tinnitus under four principle approaches: (1) learning not to think about it (for example, statement 11) or dealing with it on their own (for example, statement 47) (*n* = 9); (2) sound enrichment techniques to avoid silence and limit exposure to their tinnitus (*n* = 6); (3) reporting tinnitus to an adult (*n* = 1); (4) engagement in activities to reduce their awareness or intrusion of their tinnitus, improve their perception of the tinnitus, or create a safe place (*n* = 4).

#### 3.1.3. Clinical Assessments and Management (22 Statements)

Statements identified roles for clinicians in assessing, referring, supporting, and educating children with tinnitus and their parents in three areas: (1) tinnitus education (*n* = 4) in regard to educating both children and parents as to what tinnitus is and what reasons they may experience tinnitus; (2) referrals to ear, nose, and throat specialists and psychologists, and finding clinicians that children and parents could trust (*n* = 4); (3) the need for hearing assessments, monitoring hearing, and improving awareness of tinnitus during hearing assessments (*n* = 5).

#### 3.1.4. Support from Parents and Teachers (18 Statements, Smallest Cluster)

Statements within this cluster focussed around four themes: (1) the role of teachers and schools in supporting children with tinnitus within the school environment and allowing adjustments to exams and class routines (*n* = 7); (2) the role of parents in assisting their child, advocating for their child (*n* = 3); (3) the importance of addressing their fears and the concerns of parents (*n* = 3); (4) the role of a medical approach to tinnitus management in children (*n* = 5).

### 3.2. Rating

The mean participants' ratings of benefit from the strategies varied from 0.79, indicating a low to mild degree of benefit for one statement (*20*. *nothing, nothing special*), to 4.75, indicating a significantly high degree of benefit for three statements (*7. audiologists/clinicians are able to confidently discuss tinnitus and explain it to children and families*; *74. understand the child's needs*; *99. teach children the skills to manage their tinnitus*).

**Figure 2:**
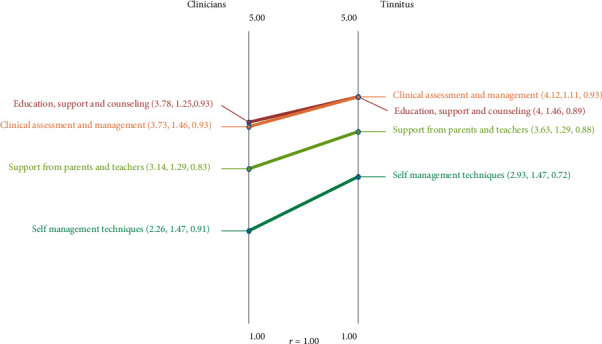
Pattern match graph comparing the tinnitus group and clinician's ratings for the degree of benefit associated with each of the clusters for “What is done to reduce the impact of chronic tinnitus on children's and adolescents' lives?” The median rating scores, standard deviations, and Cronbach's alpha scores are provided in brackets with a score ranging from 0.79 indicating a low to mild degree of benefit to 4.75 indicating between significant to highly beneficial.

In all cases, the clinicians rated the mean benefit rating of all statements in a cluster lower than the tinnitus group; this difference was significant (*p* < 0.05) for three of the four clusters (Figure 2); it was not found for the *Support from Parents and Teachers* cluster.

### 3.3. Validation and Reliability

Both the standard deviation scores and Cronbach's alpha scores associated with each cluster and each group were high suggesting that while there was individual participant variation for each statement, the internal consistency was high [37]: *Clinical Assessment and Management* (tinnitus *α* = 0.93, clinicians *α* = 0.93); *Education, Support, and Counselling* (tinnitus *α* = 0.89, clinicians *α* = 0.93); *Support from Parents and Teacher* (tinnitus *α* = 0.88, clinicians *α* = 0.83), and *Self-Management Techniques* (tinnitus *α* = 0.72, clinicians *α* = 0.91) (Figure 2).

External validation of the final concept map and concept names was achieved by presenting the four-cluster map and grouping data to all the participants. Seven participants (*n* = 4 tinnitus experiencers; *n* = 3 clinicians) responded (21% response rate), all of whom supported both the clusters derived from the data, the cluster names, and their descriptions. None requested or suggested any changes to be made.

## 4. Discussion

The purpose of this study was to identify strategies which have been used to reduce the impact of tinnitus on children who experience it from the reflections of those who experienced tinnitus during childhood, the primary carers and parents of children with tinnitus and clinicians such as audiologists, ear, nose, and throat specialists, and psychologists who care for them. During this study, participants described impact reducing strategies as occurring across four clusters: *Education, Support, and Counselling*; *Self-Management Techniques*; *Clinical Assessments and Management*; and *Support from Parents and School*. As a group, the tinnitus participants rated the statements associated with three of the clusters as more beneficial than the clinicians' group.

The benefit perceived varied from a low to mild degree of benefit for some strategies to between significantly and to highly beneficial for others. The three strategies that were associated with the greatest degree of benefit were from the *Education, Support, and Counselling* cluster: *7. audiologists/clinicians are able to confidently discuss tinnitus and explain it to children and families*, from the *Clinical Assessments and Management cluster* and statements, *74. understand the child's needs*, and *99. teach children the skills to manage their tinnitus.* These three strategies convey a regard and requirement for understanding from clinicians as well as for clinical competence and confidence. They indicate that taking time to understand what a child is experiencing is important and that being familiar and confident with discussing tinnitus and teaching strategies for managing it is reassuring to those who are affected by it.

The three statements associated with the lowest degree of benefit were all from the *Self-Management Techniques* cluster. These strategies involved either not dealing or managing the tinnitus, for example, *20. nothing, nothing special* and *47. children just deal with it* or simple avoidance strategies such as *68. children may put their fingers in their ears*. These results indicate that participants consistently regarded *Self-Management* strategies as the least helpful and that strategies from the *Education, Support, and Counselling* were the most helpful, along with *Clinical Assessments and Management*. *Support from Parents/Teachers* was regarded as helpful also but less so. These results along with the greater degree of benefit noted by the tinnitus group suggest that while children with tinnitus may develop their own coping strategies, they and their parents appreciate having the presence and possible distress of tinnitus acknowledged and value clinical knowledge, support, and guidance [15, 38, 39].

### 4.1. Education, Support, and Counselling

*Education, Support, and Counselling* was the largest cluster. Many statements within this cluster reflected a desire of participants to have children's experiences of their tinnitus acknowledged and addressed. They reflected the need to have clinicians, parents, and teachers show interest in the child's experience of tinnitus, to take time to understand each child's experience and meet their emotional needs to feel safe, supported, and reassured. Statements such as *60. listen to the child–explore impact on daily life and how they feel–how they manage tinnitus currently–what helps*, *61. provide adult acceptance so that the child knows that someone else understands*, or *52. validate the child's distress* and *39. provide child-friendly explanations for tinnitus supported with literature about tinnitus aimed specifically for children at various states to remove fear* reflect the importance of adopting a child-friendly approach to tinnitus assessment in order to facilitate effective management [15]. The concept of delivering tinnitus management in a child-friendly manner may be aligned with the tenants of patient-centred care (PCC) and family-centred care (FCC) [40]. FCC and PCC have been advocated as desirable approaches to chronic health conditions especially in paediatric health care due to the positive influence on patient outcomes [41, 42]. While PCC and FCC have been gaining increased acceptance and awareness as approaches to adult rehabilitative and paediatric hearing loss audiological care, little attention has been given to its value and application for assisting children who experience tinnitus. Sass-Lehrer (2004) and Gravel and McCaghey (2004) found that adopting a family-centred approach to paediatric hearing loss reduced stress and improved the rate of follow-up and subsequently improved patient outcomes, and Laplante-Levesque et al. (2012) reported that patients highly valued interactions where genuine interest in their needs was shown. When a child or adolescent is troubled or distressed by their tinnitus, it is likely that their parents and caregivers will be also and that the impact of tinnitus may have implications for the family as a whole [12, 43]. FCC acknowledges that when one member of a family is affected by a health condition, other members of the family may be affected also and that they play an integral role in supporting that family member in their health care [44]. Enquiring into parents' and primary caregivers' experience of tinnitus prior to tinnitus counselling and education is also important as parents or primary caregivers who have experienced tinnitus themselves may be more aware of the potential impact of tinnitus and the benefit of counselling and may be more supportive of their child's experience of tinnitus than parents who have not experienced it [13, 43, 45]. For those parents and caregivers who have not experienced tinnitus, conveying the potential seriousness of the condition is important for promoting effective support for the child outside of the clinic environment.

### 4.2. Self-Management Techniques

Statements within this cluster identified how children may independently adopt strategies for managing their tinnitus, a phenomenon previously described in the literature (Kentish [39]). Kentish [39] identified that 71% of children attending a clinic for paediatric tinnitus had independently developed strategies to manage their tinnitus prior to their first appointment, including wearing their hearing aids, listening to music/TV/radio, reading, or ignoring/distractions. The current study indicates that children may implement strategies that may be useful and effective as well as strategies that may be counterproductive such as *58. …taking recreational drugs* or *34. choose not to take prescribed medication*. Earlier studies have also identified how children with tinnitus may adopt coping strategies with counterproductive and compounding effects such as substance use and truancy [2, 43, 46]. Clinicians need to be aware that substance use may have been involved with the emergence of tinnitus [43].

It is important that clinicians are mindful of factors which may contribute to the emergence of tinnitus as well as identifying both effective, beneficial strategies and counterproductive strategies that a child may use to manage their tinnitus. Mental health concerns including depression and anxiety have also been strongly associated with experience of tinnitus during childhood and may impact on self-management strategies that children employ. The association between the experience of depression and anxiety during childhood is stronger than the association between tinnitus and hearing loss [2, 43, 46]. Concomitant anxiety or depression may impact upon a child's sense of well-being and exacerbate difficulty managing their tinnitus. Clinicians need to be aware that children may try counterproductive coping methods which may have detrimental consequences. Clinicians need to ensure that they discuss these with sensitivity and confidentiality and establish appropriate multidisciplinary supports that were required [43]. However, a recent survey undertaken exploring audiologists' knowledge of and skills for detecting and addressing their patients' mental health needs indicated that many audiologists may lack these skills [47, 48]. For clinicians working with children who experience tinnitus, developing such skills is highly recommended.

### 4.3. Clinical Assessments and Management

Statements within the *Clinical Assessments and Management* cluster outlined the role of clinicians in assessing, referring, supporting, and educating children with tinnitus and their parents. Several statements within this cluster discussed the need for hearing assessments, monitoring hearing, and improving awareness of tinnitus during hearing assessments for children. Shetye and Kennedy [15] described how clinicians are often concerned that by asking children or adolescents about tinnitus, they may create distress or worry where it previously did not exist. However, statements such as *9*. *train audiologists/clinicians to ask about tinnitus during hearing tests* suggest that the opposite is the case. Previous papers have also noted that children are often relieved to realise that they are not the only ones that experience tinnitus [15, 19, 39]. In failing to ask children about tinnitus, we deny those that experience it the opportunity for support and reassurance and instead leave them with the possibility of fear and isolation [15, 19, 20, 39].

Several statements within this cluster also addressed the issue of tinnitus education and the need to find clinicians that both the children and parents could trust. Patient education and building trusting clinical relationships are imperative for effective tinnitus management; the participants' statements emphasise the need for collaborative multidisciplinary and family-centred approaches to tinnitus management for children.

### 4.4. Support from Parents and Teachers

Statements within this cluster again reinforced the importance of a FCC approach to the management of tinnitus during childhood but from a different angle. Parents and primary caregivers are not only impacted by their child's experience of tinnitus; they are also often best placed to provide consistent support, reassurance, and advocacy when required [13]. Participants identified that the support of parents and caregivers can be both reassuring and empowering. They also highlighted the role of parents and caregivers in identifying when and how to implement strategies which address compounding difficulties.

Statements such as *98. educate and inform teachers about tinnitus and the child's need, explain that it is “real,” and educate them on how they can support the child during class and exam times* concurred with the findings of both Kentish et al. [39] and Swain et al. [12]. Children and adolescents with tinnitus often experience associated difficulties with concentration, attention, tolerance of background noise, and difficulties of hearing whether or not an accompanying hearing loss exists [39]. Children spend much of their day at school. Not only does school facilitate education, it is often central to a child's social network. As such, educating teachers regarding how to support children with tinnitus through environmental modifications, teaching strategies, and allowing children to step out when overwhelmed or to take exams in different settings can be vitally important and beneficial [12, 39].

The participants involved in this study were also involved in an earlier study where aspects of a child's life may be impacted by the experience of tinnitus (Tegg-Quinn et al. unpublished data). During that study, they identified the negative impact of tinnitus upon social interaction and participation. However, in this study, there was very little discussion or identification of strategies addressing this area of impact from tinnitus during this study. Indeed, audiologists appear to use a wide range of strategies to help their adult clients address the social impacts of hearing loss [48]. Yet, the absence of strategies addressing the social impacts of tinnitus in this study suggests that increased attention to alleviating such difficulties is required.

### 4.5. Clinical Implications

Participants of the tinnitus group indicated that that recognition of tinnitus, acknowledgement that it can cause distress, and instigating clinical care provide the basis of effective management. The higher ratings of perceived benefit associated with each of the strategies by the tinnitus group highlight the importance of acknowledging, assessing, and ameliorating tinnitus during childhood to those who are directly impacted by it. Many statements also highlighted the importance of taking time to understand each child's experience and their need to feel safe, supported, and reassured. Many statements also reflected a need for children and families to have their experiences recognised and validated. Previous papers have noted that children are often relieved to realise that they are not the only ones that experience tinnitus [15, 19, 39].

Often care for children and adolescents with tinnitus occurs within adult clinics; however, it is essential that they are not managed as mini adults [17, 49]. Statements within this study outlined the role of clinicians in assessing, referring, supporting, and educating children with tinnitus and their parents and pointed towards the importance of adopting a child-friendly approach to facilitate effective management. They reinforce the importance for both identifying tinnitus and addressing any underlying concerns, fears, and worries early to reduce associated distress [39] for clinicians to be able to discuss tinnitus, its potential causes, effects, and appropriate management techniques with confidence and to promote hope. Several statements also reinforced the importance of a multidisciplinary approach to tinnitus management with appropriate referrals to ENT specialists and psychologists.

### 4.6. Limitations and Future Research

It is possible that the views of participants within this study were informed by an experience of tinnitus that was associated with distress and which may or may not have received assistance. If this were the case, it may influence the statements that they generate and the degree of benefit they attribute to the strategies compared to a population whose tinnitus was less troublesome and thus influence the findings of this study. Additionally, recruiting adults to inform the study rather than a cohort of children currently experiencing tinnitus may also have limited the study. However, the research group was mindful of potential participant burden associated with concept mapping, basing their decision on recruitment strategy on evidence that adults can provide reliable and detailed recollections of important autobiographical childhood events and experiences that are not affected by their later experiences [34].

## 5. Conclusion

Participants within this study identified 102 strategies for managing tinnitus during childhood. The participants indicated that strategies occur within four categories: *Education, Support, and Counselling*; *Self-Management Techniques*; *Clinical Assessments and Management*; and *Support from Parents and School.* Strategies within the *Education, Support, and Counselling* and the *Clinical Assessments and Management clusters* were associated with the greatest degree of benefit. Tinnitus participants regarded efforts to ameliorate tinnitus during childhood more beneficially than the clinicians. Statements within this study also reinforced the need to ensure that tinnitus management is both age and developmentally appropriate. Participants also identified that while many children will develop strategies to assist with their tinnitus themselves, strategies associated with clinical assessment, knowledge, and expertise were regarded more highly. Having clinicians ask about tinnitus during hearing assessments and to be able to discuss tinnitus, its potential causes, effects, and appropriate management techniques with confidence and to promote hope were identified as important and effective strategies for reducing the impact of tinnitus upon children. Such approaches would be enhanced when care is provided in a FCC approach where parents and primary caregivers are also provided with tinnitus education and strategies for supporting their children.

## Figures and Tables

**Table 1:** Overview of concept mapping stages and participants.

Stage	Description	Method of participation	Tinnitus group	Clinician group	Participating research team members
Brainstorming	Participants produced statements in response to the question “What is done to reduce the impact of chronic tinnitus on children's and adolescents' lives?”	Entered directly into online portal or transcribed during teleconference. No participant chose the face-to-face brainstorming session.	*N* = 20	*N* = 12	ST-Q
Statement pooling and refining	The participants' statements were pooled, reviewed, and refined. The research team removed duplicate and irrelevant statements and edited the remaining statements to produce clear, nonidentifiable, and pertinent statements.				ST-Q, RB, RE
Grouping	Participants grouped the statements in a manner that made sense to them. The resulting groupings assisted identification of common themes.	Directly undertaken in online portal or via post pack	11	13	ST-Q
Rating	Participants rated each statement using a 6-point Likert scale according to degree of benefit associated with each statement: 0 = low degree of benefit, 1 = mildly beneficial, 2 = minor degree of benefit, 3 = moderately beneficial, 4 = significantly beneficial, and 5 = highly beneficial.	Directly undertaken in online portal or via post pack	11	13	ST-Q
Data analysis and interpretation	A point map was produced from multidimensional scaling. How closely the points were to one another reflected how frequently the participants grouped the statements together. Reliability of the point map was established through calculating a stress index. Hierarchical cluster analysis produced a cluster map highlighting key concepts. Each cluster represents a concept (Figure 1). Consistency of the participants' grouping and rating data was established through calculating split-half reliability tests. Reliability of the participants' rating data was established through calculating Cronbach's alpha. Cohort comparisons were facilitated through generation of a pattern matching graph. The cluster map was presented back to the participants and seeking their feedback on the clusters, the cluster names, and their descriptions for external validation	Concept Systems software and SPSS			ST-Q, RE

## Data Availability

Participant rating data is available by emailing the first author susan@hearelief.com.
