# Supplementary material for: Reducing the Impact of Tinnitus on Children and Adolescents' Lives: A Mixed-Methods Concept Mapping Study
Source: Int J Pediatr. 2021 May 31;2021:5534192. doi: 10.1155/2021/5534192 (PMC8187041; doi:10.1155/2021/5534192)
Supplement: Supplementary Materials — Cluster statements are available in the supplementary file attached to this article. [file 5534192.f1.docx]

**Cluster Statement**

1. **Education, Support & Counselling**
2. Counselling with family as to why tinnitus has become a problem.

4 Learn what triggers the child’s tinnitus and discuss the triggers.

5 Challenge the stereotype that tinnitus is only experienced by old men with hearing loss.

10 Encourage child to become aware that there are times when the tinnitus is not there so

that they learn to become less aware of it.

13 Talk about it when it is really unbearable.

16 Teach children how not to think about the tinnitus (Learning to control it without other

people noticing).

19 Educate children about tinnitus.

23 Work out what meaning has been associated with the sound.

25 Assist child to manage their stress levels.

27 Reduce fear.

28 Attend tinnitus support days.

29 Provide children who have tinnitus with lots of encouragement and hope.

31 Holistic approach to addressing situations where tinnitus is a problem so that the child is

supported across all environments.

36 Teach children relaxation techniques.

37 Assist children learn that they are safe.

38 Know that there is no quick fix available.

39 Provide child friendly explanations of tinnitus supported with literature about tinnitus

aimed specifically for children at various stages to remove fear

52 Validate the child’s distress.

58 Inform children that taking recreational drugs can make tinnitus worse.

60 Listen to the child – explore impact on daily life and how they feel – how they manage

tinnitus currently – what helps.

61 Provide adult acceptance so that the child knows that someone else understands – they

will talk more about it.

63 Provide an alternative way of thinking about the noise e.g. “the noise of the stars at

night”.

69 Remove visual stimulation and screen time before bed.

70 Turn the negative experience of tinnitus into a positive, the child can become and

advocate for paediatric tinnitus. (Empowerment)

74 Understand the child’s needs.

75 Give hope that there will be better treatments in the future.

77 Provide tolerance for activities that a child may not be able to take part in because of

their tinnitus.

79 Remain positive give cautiously optimistic prognosis and don’t use terms like

permanent.

83 Provide support, support, support.

84 Reassure the child.

88 Practical steps to minimise perceptions of tinnitus.

89 Have management plans developed in collaboration with the child that involve

strategies for various situations e.g. in class or at home. Start with what they already

notice is helpful, build confidence in how they can move attention away from tinnitus.

90 Identify with characters in popular media with tinnitus (e.g. Lead character of a recent

movie who developed tinnitus after a car accident).

93 Regularly try to decrease the awareness of Tinnitus by exposing the child/adolescent to

various typical ambient noises. Such as water falling, rain, crickets ….. The stress levels

will come down and the noise will be disguised.

96 Find other children with tinnitus to talk with or write to. (So that they realise that they

are not the only child with tinnitus)

99 Teach children the skills to manage their tinnitus.

1. **Support From Parents & Teachers**
2. Encourage parents to stop asking their child about the tinnitus to reduce drawing

attention to it.

15 Schools provide support and understanding of the condition and the child’s

requirements.

17 Parents seek help on behalf of the child.

24 Children with tinnitus need classes that are ordered – they are easier for them to follow.

26 Inquire as to whether there is a school counsellor who may be helpful.

32 Provoke/inspire patient organisations to take an interest in the area.

35 Go to the doctor.

53 A child may be prescribed medications. (e.g. sleeping tablets, tranquilisers, aspirin)

55 Other family members who experience tinnitus can normalise the experience of tinnitus,

provide support and lessen fear.

57 Parents may give their child an explanation about the cause of the tinnitus, it may not be

accurate.

78 What is done depends on how responsive the doctor is – how ready they are to believe

the child.

85 Teachers allow the child to leave class when they become overwhelmed.

87 Raise awareness of condition with teachers.

95 School allows children with tinnitus to take exams at a different time or in a different

environment.

97 Address parents’ concerns and fears.

98 Educate and inform teachers about tinnitus and the child’s needs, explain that it is “real”

and educate them on how they can support the child during class and exam times.

100 Raise awareness of condition with caregivers.

101 A child may be hospitalised.

1. **Clinical Assessments & Management**

6 Undertake speech overload testing.

7 Audiologists/clinicians are able to confidently discuss tinnitus and explain it to children

and families.

9 Train audiologists/clinicians to ask about tinnitus during hearing tests.

18 Involve a paediatrician in the child’s care.

40 Address ear nose and throat issues.

41 Using other test results to identify when to ask a child about tinnitus. When this occurs

it is often the first time that a child reports that they have noises and the first time their

parents become aware.

42 Undertake test to determine what it means.

44 Refer children with tinnitus to an Ear Nose and Throat specialist to exclude possible

underlying conditions.

46 Provoke/inspire clinicians to take an interest in the area.

49 Improve access to hearing tests.

50 Find people (clinicians) that the child and parent can trust.

56 Hearing tests to confirm normal peripheral hearing and provide hearing aids as soon as

possible if needed.

64 Refer child to psychologist to address anxiety.

67 Find the correct cause of the tinnitus.

71 Identify if other problems are present also e.g. hyperacusis (where sounds feel

uncomfortably loud) or misophonia (where some sounds spark a strong emotional

response)

73 Provide children and parents with reasons why tinnitus may be experienced (health,

medications, stress, changes in diet, ear wax accumulation and exposure to loud noises

etc.)

76 Monitor children’s hearing levels when there has been a history of noise exposure.

80 Educate about hearing, the effects of noise exposure and the importance of hearing

protection.

81 Normalise the experience of tinnitus for the child and parents.

91 Treat co-incident hyperacusis.

92 Use clinic supports to relay information to children and parents.

94 Assist and educate parents.

1. **Self-Management Techniques**
2. Children may make extra noise to cover their tinnitus.

8 Try to face tinnitus in pure silence. Recurrent activities like reading literature, painting,

yoga (activities that require a low amount of noise), can actually transform Tinnitus into

something as usual as silence itself. The body perceives change.

11 Learn not to think about it. (child learns on their own)

12 Children reporting it to their parents.

14 Use an FM radio band and not white noise for masking the tinnitus noise.

20 Nothing, nothing special.

21 Play computer games to keep mind off tinnitus.

22 Sound enrichment. (have noise or music on in the background)

30 Sleep with a calming sensory LED light projector on.

33 Put music on at night.

34 A child my chose not to take prescribed medications. (e.g. sleeping tablets)

43 Improve sleep through the use of a bedside environmental sound generator.

45 Learn to distinguish the tinnitus from other sounds.

47 Children just deal with it.

48 Carry ear plugs and headphones and use some tracks (with ambient noises) to disguise

and reduce awareness of Tinnitus, during peaks.

51 Children can develop their own safe space.

54 Reading existentialist literature may improve perception of body condition. Camus,

Satre, Beauvoir

59 Wear white noise generators.

62 Learn to just ignore it.

65 Children manage their tinnitus on their own.

66 Many children just put up with it.

68 Children may put their fingers in their ears.

72 Learn how not to listen to it. (Child learns on their own)

82 Children use a combination of self-regulation and avoiding tinnitus triggers.

86 Avoid silence.

102 Reducing the impact of tinnitus can be an unconscious process for many children.

|  |  |  |  |  |  |  |  |  |  |  |
| --- | --- | --- | --- | --- | --- | --- | --- | --- | --- | --- |
